# Annotations

**Published:** 1897-03-13

**Authors:** 


### Annotations.

### Schools and Diphtheria.

In regard to the very important question of the part taken by the elementary schools in the spread of diphtheria, which was discussed in our issue of February 27th, Professor W. R. Smith has forwarded to us a report on the subject which has been drawn up by him for presentation to the London School Board. He has made a very careful analysis of the cases with the result of showing how difficult it is to trace the spread of infection from case to case. We doubt, however, whether it would be wise to acquit the schools entirely on such grounds, for the broad facts remain much as they were stated by Dr. Hamer in his report. Professor Smith has, we think, good reason on his side in insisting on the importance of the sanitary condition of the home in the etiology of diphtheria. The tendency which has lately shown itself among certain sanitarians to minimise the share taken by insanitary conditions in the production of diphtheria is, we think, founded on a narrow view of the subject. Nevertheless, the final act in the catching of an infectious disease is the reception of the infection, and we cannot but accept the statistics which have again and again been put forward to show that the schools are at least one mode by which diphtheria is spread. In fact, Professor Smith practically admits this when he says, "The comparative escape of the girls' department is in part attributed to the special care exercised by the head-mistress in the exclusion of all suspicious cases," and "I admit that some of the twenty-one cases of unknown origin were possibly due to infection in school, but I feel certain that if all the suspicious cases of sore throat had been carefully excluded hardly a single case of infection would have occurred in the Board schools." The object of Professor Smith's report is to show that it was unnecessary to close the schools. That is a point on which we need not here touch; but at least we may take it that his anxiety to exclude suspicious sore throats is an admission that they are sources of infection when they are admitted, as they must very often be.

### Is Plague Contagious?

At the "Plague" Sanitary Conference the other day, Dr. Cleghorn, the British representative from India, speaking of plague, declared that as yet there were few scientific data available which were absolutely trustworthy. Then he affirmed that most of the really competent authorities in India were inclined to question the purely "contagious" character of plague. It seemed to them to be a disease "mainly of local conditions." To that we should unhesitatingly give an assent. For long we have doubted the evidence which seemed to show that a blood-poison disease like plague could be transmitted from person to person merely by contact of external parts. All the experience which was gained in Hong Kong goes against it, and the idea of transmission by external contact is, apparently at any rate, as "unscientific" as it can well be. Dr. Cleghorn thinks that neither in this country nor in Southern Europe need there be any undue alarm at the prospect of the plague approaching. For our part we feel assured that in England and Europe we have the matter largely in our own hands. Let us surround ourselves with all the conditions of health which science can so well help us to; and then we may, with reassured minds, bid to every form of "contagion" a long defiance.

### Our Colour Blind Seamen.

How far it is justifiable to do a grave injury to an individual for the public good is just one of those questions which casuists delight to argue. In the abstract we have no doubt that the public safety must always be the first consideration, but we are equally clear that in the application of this general principle private interests should be safeguarded, and hardship to the individual should be prevented as far as possible. The safety of the travelling public, nay, also of seamen as a class, undoubtedly demands that sailors should have perfect vision. If this be granted—and who will not grant so much?—it is difficult to gainsay the proposition that the examination of their eyes, an examination on which their livelihood depends, should be made by medical experts whose experience would enable them to give a mature and reliable judgment on the subject, and that it should be made at the time when they are entering on their career, and not when, after years of work and drudgery, and when they have become unfitted for any other form of occupation, they apply for promotion to the rank of second mate. Yet in England, for all our boasted love of fair play, neither of these things is done. Mr. Bickerton, of Liverpool, writing on this subject, in a proper vein of indignation, says: "Allowed to enter, without let or hindrance, the sea profession, actuated by the best wishes and intentions to rise in the service selected by them for their life's work, they have passed through the drudgery and hardship common to a sailor's lot, and now, after three, four, five, six, or seven years—as the case may be—enter for their second mate's certificate and are told they are colour blind." It is difficult to imagine a greater hardship than this, or anything more likely to bring the mercantile marine and all its ways into disrepute among the classes from whom our sailors ought to be recruited. This, however, is not all. The examination itself is not conducted by experts, nor does it appear that even those to whom the duty is now deputed are provided with proper rooms and apparatus for the purpose. Thus is a double injustice done to these unfortunate men. The extent of this injustice may be judged of when it is understood that a considerable number of those whose circumstances allow them to appeal against the sentence of these unskilled examiners succeed in obtaining a reversal of the judgment. Of 101 rejected candidates we are told that 21 appealed, and of these 21 individuals no less than eight succeeded. That is, the judgment of the examiners was reversed in 38 per cent. of those who appealed! We should be the last to wish that men with defective eyesight should become officers in our mercantile marine, but we maintain that they should be stopped at the beginning and not in the middle of their career, and that the examination, on which their living depends, should be made by experts who, at least, would not make 38 per cent. of errors.

